# Ocular characteristics in a variant microcephalic primordial dwarfism type II

**DOI:** 10.1186/s12887-019-1685-2

**Published:** 2019-09-11

**Authors:** Wan-Ju Chen, Fu-Chin Huang, Min-Hsiu Shih

**Affiliations:** Department of Ophthalmology, National Cheng Kung University Hospital, College of Medicine, National Cheng Kung University, 138 Sheng Li Road, Tainan, 704 Taiwan, Republic of China

**Keywords:** Microcephalic osteodysplastic primordial dwarfism type II (MOPD II), Pericentrin (PCNT) gene, Macular scar, Hyperopia

## Abstract

**Background:**

Microcephalic osteodysplastic primordial dwarfism, type II (MOPD II) is a rare disease that is assumed to be caused by a pericentrin (PCNT) gene mutation. Clinical manifestations have been reported in pediatrics and neurology; however, only a few ocular findings have been documented.

**Case presentation:**

We present three unrelated cases of MOPD II with similar facial features and short stature. Unlike the cases described in the literature, all subjects had normal birth weight and height but their growth was retarded thereafter. In addition to delayed milestones, they have a broad forehead, maxillary protrusion, long peaked nose, high nasal bridge, low-set large ears, extreme reromicrogenia, and normal-sized teeth. These three patients had similar ocular manifestations with the short axial length associated with high hyperopia more than + 9 diopters (D) and macular scarring. The oldest subject was a 20 year-old male without neurological symptoms. One female subject had developed alopecia during the previous 2 years. The other female subject had moyamoya disease, but a genetic study revealed a normal PCNT gene.

**Conclusion:**

This is the first report of MOPD II focusing on ocular findings, suggesting that macular dystrophy and high hyperopia are the common ocular characteristics of MOPD II. Prompt referral to an ophthalmologist is essential. Although refractive amblyopia can be treated with optical correction, visual prognosis may be poor due to maculopathy.

## Background

Microcephalic primordial dwarfism comprises a group of rare disorders characterized by extreme growth failure [1]. Microcephalic osteodysplastic primordial dwarfism, type II (MOPD II) is the most common type, for which the clinical characteristics have been well described. It has been demonstrated that mutations in the pericentrin (PCNT) gene are associated with MOPD II [2, 3]. The PCNT gene, located on 21q22.3, encodes the PCNT protein. Dysfunction of the PCNT protein leads to disorganized mitotic spindles and missegregation of chromosomes [4], which affects cell division during growth. Diagnosis of MOPD II is usually made clinically based on the typical features although a molecular analysis of PCNT is an alternative tool to confirm the diagnosis in some cases [5]. Herein, we report three isolated cases clinically diagnosed as MOPD II sharing similar ophthalmic features. Data regarding cycloplegic refraction, best corrected visual acuity, a slit lamp examination, ocular coherence tomography (OCT), and fundus photography are recorded.

## Case presentation

### Case 1

This male patient presented at our clinic due to nystagmus and deviated eyes when he was 5 years old. He was term and born at a 3655 g body weight. However, failure to thrive was noted after he was 2 years-old. Serial endocrine levels were checked and indicated normal data. No family history was reported.

**Fig. 1:**
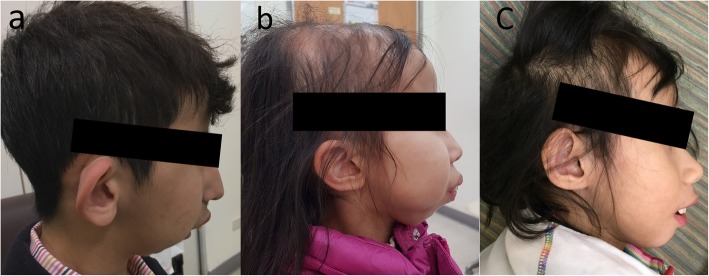
**a-c** The characteristic face for the three cases. (**a**, Case 1; **b** Case 2; **c**, Case 3)

**Fig. 2:**
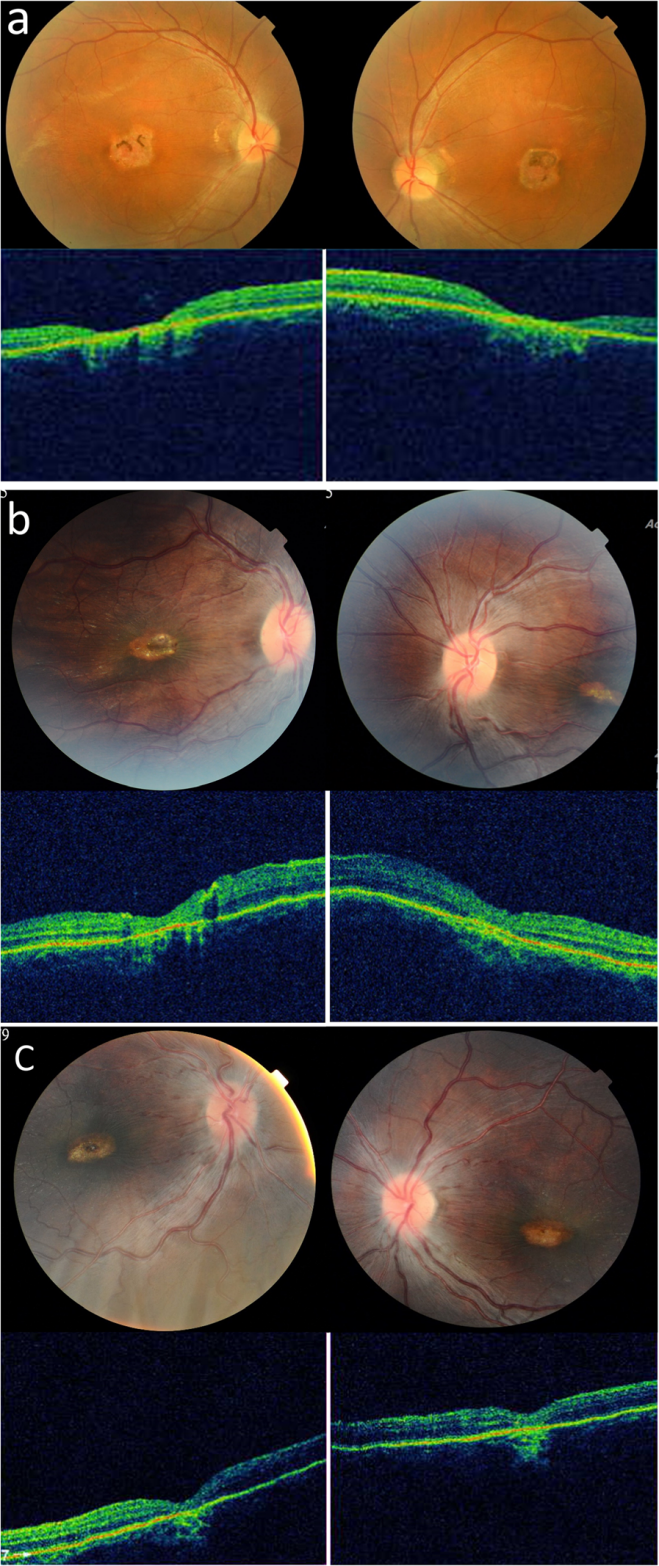
The macular scar in the three cases, shown by fundus photography and ocular coherence tomography (OCT). (**a**, Case 1; **b** Case 2; **c**, Case 3)

The initial ophthalmologic exam revealed high hyperopia with + 9.0 diopters (D) in the right eye and + 8.5 D in the left eye and high astigmatism − 3.0 D in both eyes. Best corrected visual acuity (BCVA) was 20/200 in the right eye and 20/60 in the left eye. Exotropia of approximately 25 prism diopters (PD) was found in the right eye. Slit-lamp biomicroscopy revealed no abnormal findings, but an ophthalmoscope examination showed macular scarring in both eyes. After spectacle correction for 5 years, his BCVA was improved to 20/60 in the right eye and 20/30 in the left eye. The amplitude of nystagmus was decreased. The patient received regular follow-up at our department for more than 10 years. When he was 20 years old, he had an extremely short stature, with a body height of 117.5 cm and a weight of 24.5 kg, bone dysplasia, and facial characteristics including a receding chin and prominent ears (Fig. 1a). High hyperopia and astigmatism were still present, and the axial length was 20.28 mm in the right eye and 20.37 mm in the left eye. Optical coherence tomography (OCT) confirmed an atrophic retina in the macula area (Fig. 2a).

### Case 2

This female patient visited the ophthalmologic clinic due to exodeivation and nystagmus at 5 years of age. She was born full term with a birth weight of 2540 g. Endocrine and genetic surveys did not indicate abnormalities. Amblyopia resulting from high hyperopia and astigmatism was found. A prism cover test revealed 40 PD of exotropia. An oval scar was found at the binocular macula (Fig. 2b). She received optical correction at that time. When she was 15 years old, her body weight was 19.8 kg, and her height was 112.3 cm. She had normal intelligence, and her corrected visual acuity was 30/200 in both eyes. Hyperopia + 11.0 D and astigmatism − 3.0 D was still noted, with an axial length of 18.44 mm in the right eye and 18.35 mm in the left eye. In addition to the characteristic facial features and short stature, hair loss and alopecia had occurred since she was 10 years of age (Fig. 1b).

### Case 3

**Fig. 3:**
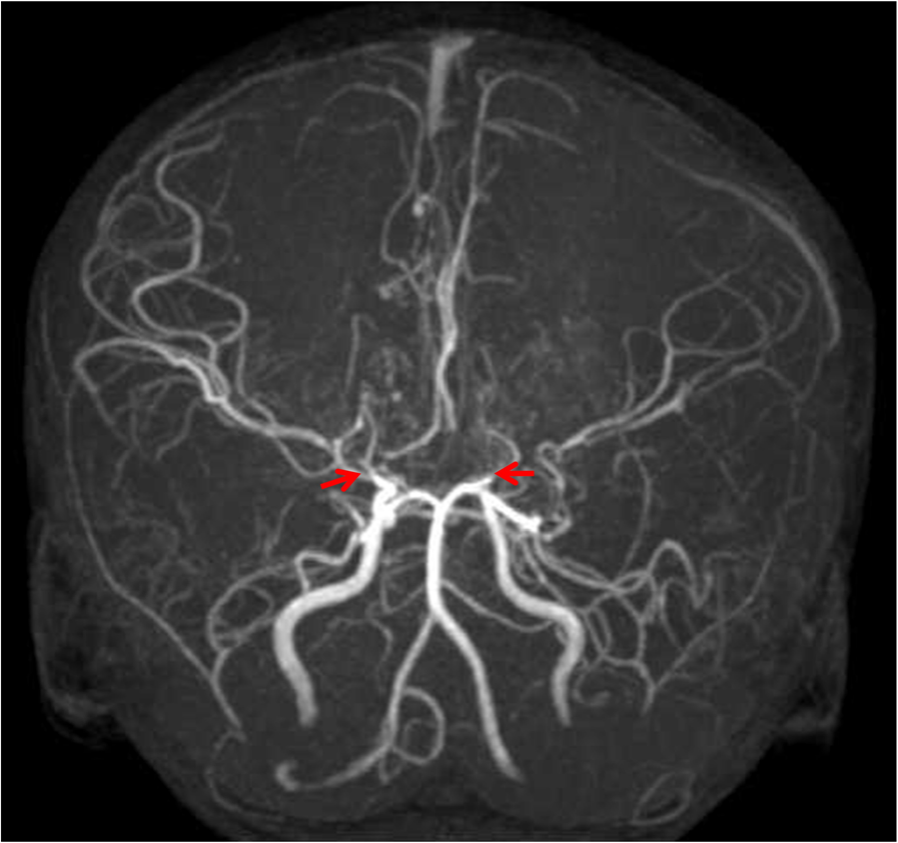
Moyamoya appearance in case 3. (obliteration of the bilateral supraclinoid internal carotid arteries pointed by arrow)

This 5 year-old female child was born term with a birth body weight of 3000 g. However, developmental delays and failure to thrive were noted when she was 2 years old. She was sent to the hospital due to sudden right upper limb weakness after waking in the morning. During the episode, she had clear consciousness, good spirit, and a good appetite. A neuroimage revealed a recent infarct with acute cytotoxic edema at the left frontal lobe and right centrum semiovale. Magnetic resonance angiography (MRA) disclosed an obliteration of the bilateral supraclinoid internal carotid arteries (ICAs) with adjacent net-like vessels proliferations consistent with moyamoya disease (Fig. 3). Due to her dysmorphic face (Fig. 1c) and short stature, a chromosomal study was arranged that showed a normal karyotype. A serial endocrine assay revealed no abnormal findings.

She was referred to the ophthalmologic department for a vision examination because she looked at near objects at a very short distance. On the first examination, the BCVA was 20/200 in both eyes. High hyperopia was noted with + 9.75 D in both eyes. A fundus examination revealed maculopathy in both eyes (Fig. 2c), which was confirmed by OCT. Optical correction with spectacles was prescribed for treatment of refractive amblyopia. When she was 6 years old, her body height was 101 cm. The axial length was 18.13 mm in the right eye and 18.01 mm in the left eye. She and parents had received molecular genetic analysis of the PCNT gene, but no mutation was noted.

## Discussion

MOPD II is an autosomal-recessive disorder characterized by microcephaly, short stature, characteristic facial features, and abnormal dentition [5–7]. Based on the facial characteristics described in the literature [5, 7, 8], these three unconsanguineous patients had a similar bird-headed facial appearance with a broad, receding forehead, long peaked nose, high nasal bridge, maxillary protrusion, low-set prominent ears, extreme retromicrogenia, and normal-sized teeth, suggesting that they were experiencing the same disease. They also presented the same ocular manifestations, with the maculopathy and extremely short axial length associated high hyperopia. They were the only members of their family with the condition. The cases discussed here were classified as a variant of MOPD 2 because they didn’t have intrauterine growth retardation. Therefore, a higher body height was noted compared to the previous cases presented in the literature [5, 6, 9]. In addition, they all had normal size teeth and normal intelligence, which was different from the cases discussed in the literature [6, 7, 10, 11].

The previous two cases did not have neurological symptoms and signs until the present time. Case 1 was an adult, which is rare in MOPD II^5^. Case 2 had acquired alopecia, which was not present in the other two cases. Case 3 had moyamoya disease resulting in one episode of a cerebral infarct, which was compatible with an MOPD II diagnosis. The association of moyamoya disease and MOPD II has been well demonstrated [12–14]. Waldron et al. also revealed that 25% of patients with MOPD II have intracranial moyamoya and aneurysms [13]. In addition, Bober et al. followed 25 MOPD II patients undergoing neurovascular screening tests, and found that 13 (52%) of the patients had moyamoya and/ or intracranial aneurysms [14]. Moyamoya disease results from occlusion of the blood vessels due to extensive fibrocellular intimal thickening [15] . It starts from the stenosis of the large arteries, which is progressive and subsequently stimulates compensated growth of small collateral vessels with a “puff-of-smoke” appearance in angiography, the so-called “moyamoya disease.” These cerebrovascular problems expose MOPD II patients to a higher risk of stroke [14, 16], as was the case with case 3. Thrombolytic therapy is not recommended in moyamoya patients with only one stroke episode since hemodynamic problems rather than thrombus formation and embolization pose a risk of hemorrhage in areas of moyamoya collateral vessels [17]. The parent of this child was concerned about the risk and refused embolization.

Ocular manifestations in MOPD II have rarely been reported. Far-sightedness has been found in some patients with MOPD II [4]. However, there is a paucity of studies addressing ophthalmological findings in these patients. Bang and colleagues described “ocular moyamoya” in a 5 year-old boy with MOPD II presenting unilateral cerebral vascular moyamoya disease and ipsilateral iris collateral vessels [18]. There are two cases in the literature documented with abnormal retinal pigmentation, one with a macular scar and the other one with retinal vascular changes [7]. In our study, all of the subjects had atrophic macular scarring and normal retinal pigmentation. The mechanism of macular scarring in these patients is still unknown. During long-term follow-up, Case 1 had mild change in the pigmentation of the macular scar.

Case 3 and her parents had received a genetic analysis of the PCNT gene, but there were no positive result yields even after conducting a whole genomic study. Unlike the previously presented cases [5], these three children had normal birth weight and normal sized teeth. Mutations other than PCNT gene might be the reason for their symptoms although they were diagnosed clinically as having MOPD II.

## Conclusions

Infantile nystagmus is usually related to a wide variety of ophthalmological or systemic disorders. Prompt referral to ophthalmologists for children with nystagmus or developmental retardation cannot be overemphasized. Refractive amblyopia can be treated with early optical correction for high hyperopia resulting from an extremely short axial length despite maculopathy.

## Data Availability

All data generated or analyzed during this study are included in this published article.

## Abbreviations

BCVA: Best-corrected visual acuity
MOPD II: Microcephalic osteodysplastic primordial dwarfismtype II
OCT: Optical coherence tomography
PCNT: Pericentrin
